# 

**Published:** 1895-09-28

**Authors:** 


**Notices of Births, Deaths, and Marriages** are inserted in this column at a charge of 2s. 6d. for an announcement not exceeding four lines.

**Notice to Advertisers and Subscribers.**

All Advertisements, Orders for Copies of The Hospital and Business Communications should be addressed to **The Manager, NOT TO THE EDITOR**, The Hospital, 428, Strand, London, W.C.

The Cost of Subscription, post free, is as follows:—Per week, 2½d.; per quarter, 2s. 8d.; per half-year, 5s. 3d.; per annum, 10s. 6d. Foreign:—Per Annum, 15s. 2d.; payable, in all cases, in advance.

NOTICE TO CORRESPONDENTS.

All MS., Letters, Books for Review, and other matters intended for the Editor should be addressed The Editor, The Lodge, Porchester Square, London, W.

The Editor cannot undertake to return rejected MS., even when accompanied by stamped directed envelope.

**"Burdett's Hospital Annual," 1895,**

Sixth year of publication. 950 pp. Scarlet cloth, gilt lettered. Price 5s. A most complete guide to British, Colonial, Indian, and American Hospitals, Dispensaries, Nursing and Convalescent Institutions, and Asylums. A few copies of the "Annual" for 1894 may still be obtained on application to the Publishers, The Scientific Press (Limited), 428, Strand, London, W.C.

**NOTICE.—Vol. XVII. of "The Hospital" (October, 1894, to April, 1895), in handsome cloth binding, is on sale at the Office of this Journal. Price 6s. Cases for binding the Numbers contained in this Volume, 1s. 6d. Index to Vol. XVII., 2½d., post free.**

**The Monthly Part for October will be ready on October 1st, price 6d.; by post, 8d.**

### SCRAPS AND GLEANINGS.

A FETE is to be held this week at Longton to assist the funds of the Cottage Hospital.

DURING last year 14 seamen and six cargo labourers had been treated in the Cornelia Hospital at Poole.

A CARNIVAL and concert has been held at Brierley Hall, Birmingham, in aid of the Foresters' Convalescent Home at Clent.

THE committee of the Midland Counties Oddfellows' Convalescent Home are to be congratulated on the improved state of their finances.

AN anonymous donor has presented the convalescent home of the Deptford Medical Mission with £100. The Mary Ward is the outcome of this donation.

THE result of the Hospital Saturday collection at Brighton was a splendid success this year, over £1,028 having been raised in aid of the local charities.

ONE thousand pounds has been left the Grimsby Hospital by Mr Soames, and the board consider that a portion of it had best be applied in the scheme of alterations.

IN common with kindred associations in Sussex, the Portslade friendly societies had an annual Church parade. The collection this year was made in favour of the funds of the Brighton medical charities.

THE New York *Medical Times* remarks: "It is claimed that at least 10 per cent of the patients in the chief dispensary of the city suffer from tea drunkenness; its effects are noticed primarily in connection with the nervous system."

THE Local Government Board Inquiry into the application of the Richmond Town Council for sanction to a loan of £5,000 for the purchase of a site for an isolation hospital at Mogden, in the parish of Isleworth, has just taken place.

THE Linden Convalescent Home at Blackrock is badly in need of funds. Last week a Viceregal visit was paid to it, and the wards and the grounds attached to the home were duly inspected. Linden is the only Catholic convalescent home in Dublin, and is under the care of Sisters of Charity.

THE Tonbridge Hospital Sunday Demonstration organised by the various friendly societies was this year on a far grander and more gigantic scale than its predecessors. The demonstration is held in aid of the General, the Eye and Ear, and the Homœopathic Hospitals at Tunbridge Wells.

TOWARDS the estimated cost of £767 for the children's ward at the Bideford and District Infirmary, the committee had on the opening day a deposit of £209—a gift from Mr. Oliver of £100, which, together with various other subscriptions, leaves only £66 still to be raised for this excellent object.

THE Colonial Secretary for South Africa is carrying into effect his promise made to Parliament with regard to the recommendation of the Leper Commission. The reforms thus to be introduced include the establishment of licensed leper houses on the mainland, to be conducted under strict rules and close medical supervision.

THE report of the Dundee Royal Infirmary presented last week shows that there is still difficulty in getting ends to meet for the future in connection with the institution. It has been pointed out that the working masons, joiners, and other craftsmen might arrange an organised system of united subscription to the funds of the institution.

SERIOUS financial difficulties are being experienced at the Ashford Cottage Hospital. The present committee is doing everything in its power to diminish expenditure so far as is compatible with efficiency, and during 1894 more good work was done, and, by special efforts, a larger income was obtained than in any preceding year.

THE last report of this institution referred to the growing need of some special provision for children, and that want must have continued unsatisfied had not Mr. Corbett's generosity again been displayed. He has given £500 to provide the requisite additions to the buildings and furniture, and also £1,000 for the endowment of the children's ward.

THE annual report of the North-Eastern Hospital for Children, which has very recently been presented, shows that the institution is doing valuable work among the poor, a work which is efficiently being carried on in spite of insufficient funds and heavy debt. The committee are appealing to the public for support in their efforts to remove these disadvantages.

THE authorities of the Belfast Royal Hospital are considering ways and means of raising their subscription list, which shows a decided lessening. On a total of twelve months the committee report being nearly £1,000 to the bad, a state of things which certainly is not creditable, considering that the claims of the institution are increasing, and also bearing in mind the broad character of the institution's work.

THE annual report of the Corbett Hospital, Stourbridge, Worcestershire, states that the experience of another year has abundantly justified the generosity which led to the establishment of the institution. Not only have the eighteen beds set up twelve months ago been regularly occupied, but increasing demands have led to the addition of several new ones; even now it is not always possible to admit all applicants.

A MEETING of owners and masters of ships has just been held in the Seamen's Institute, Poole, for the purpose of considering a scheme for providing hospital aid and attendance for any sick or injured seamen. The question was discussed whether it would not be advisable to start a scheme by which a master or owner of a vessel entering the harbour should be asked to pay a voluntary rate in order to create a fund to provide this said aid.

THE committee of the Salisbury Infirmary are again compelled to draw attention most forcibly to the inadequacy of the income of the infirmary to meet its annually increasing expenditure and requirements, and while they are strongly urged, on the one hand, to embark in the building of a nurses' home, they hesitate to add, on the other hand, to the establishment charges which would thereby inevitably be increased. It is calculated that the average expenditure is now at least £300 in excess of income. The anxiety which exists for the establishment of a home for the nurses is shown by the action of Mr. S. Jones, who in August last sent a donation of £100 to the fund for building the nurses' home at the Salisbury Infirmary, and has now forwarded to the treasurer a similar amount for the same purpose.

### BOOKS RECEIVED.

KEGAN PAUL.

"The Two Thrones." By John A. Goodchild.

SWAN SONNENSCHEIN.
"Analytical Key to the Natural Orders of Flowering Plants." By Franz Thonner.

F. J. REBMAN.
"Law and Chemistry of Food and Drugs." By Robinson and Cribb.

SIMPKIN AND MARSHALL.
"The Woman who Wouldn't." Lucas Cleeve.

**Periodicals and Pamphlets.**—The Humanitarian, Cornhill Gentleman's Magazine, Cassell's Family, Public Health, The Asclepiad, Monthly Homœopathic Review, Revue Générale des Sciences, Westminster Review.

### The Student of Medicine.

### APPOINTMENTS.

E. W. Battle, M.R.C.S.Eng., L.R.C.P.Lond., appointed House Surgeon to the Manchester Royal Infirmary. C. K. Bruce, M.B., M.C.Edin., appointed Senior House Surgeon to the Cumberland Infirmary, Carlisle. A. E. L. Charpentier, M.D.Durh., L.R.C.P.Lond., appointed Medical Officer for the Uxbridge District of the Uxbridge Union. Dr. Clarke, appointed Medical Officer for the Aston District of the Rotherham Union. R. D. Cran, M.R.C.S.Eng., L.R.C.P.Lond., appointed House Surgeon to the Manchester Royal Infirmary. W. E. Dawson, L.R.C.P.I., L.M., L.S.A., appointed Admiralty Surgeon for Walton-on-the-Naze and Handford Waters. W. Doughty, L.R.C.P., L.R.C.S. Edin., appointed Medical Officer for the No. 7 District of the Kingsbridge Union. W. O. Evans, L.R.C.P., L.R.C.S.Edin., appointed Medical Officer for the No. 1 District of the Hawarden Union. J. Greenhalgh, L.R.C.P.I., L.M., appointed Medical Officer for the Fylinglaes District of the Whitby Union. J. P. Hall, M.B., Ch.B.Vict., M.R.C.S.Eng., L.R.C.P.Lond., appointed House Surgeon to the Manchester Royal Infirmaoy. G. R. Harcourt, M.R.C.S., L.R.C.P.Lond., appointed Assistant Medical Officer at the Lambeth Union Infirmary. R. J. Hillier, M.R.C.S., L.R.C.P.Lond., appointed Senior House Surgeon to the West Ham Hospital. J. D. Kenny, M.D.R.U.I., M.Ch., appointed Medical Officer for the Treeton District of the Rotherham Union. T. Linde, M.D.Edin., appointed Medical Officer for the No. 5 District of the Westbury-on-Severn Union. A. H. McDougall, M.R.C.S., L.R.C.P.Lond., appointed Medical Officer of the Birmingham Parish Workhouse. L. J. Minter, M.D. Brux., M.R.C.S., appointed Medical Officer to the Uxbridge Union Workhouse. F. C. Moore, M.Sc., M.B., Ch.B.Vict., appointed House Surgeon to the Manchester Royal Infirmary. H. F. Nunes, M.R.C.S., L.R.C.P.Lond., appointed Medical Officer of the Clun District of the Clun Union. Dr. G. W. Pickering, appointed Medical Officer for the No. 9 District of the Hexham Union. W. A. Pinniger, M.R.C.S., L.R.C.P.Lond., appointed Medical Officer for the No. 6 District of the Brighton, Hove, and Preston Provident Dispensary. E. B. Roberts, L.R.C.P., L.R.C.S.Edin., appointed Medical Officer for the No. 1A District of the Hawarden Union. M. Sharman, M.B.Glasg., M.R.C.S.Eng., appointed Divisional Surgeon to the Herts County Constabulary. C. Spurrell, F.R.C.S.Edin., L.R.C.P. Lond., appointed Medical Superintendent to the Poplar and Stepney District Sick Asylum. R. H. Steen, M.B.Lond., appointed House Physician to St. Mary's Hospital, W. J. A. Swainson, M.B.Cantab., L.R.C.P., L.R.C.S., L.M.Edin., L.F.P.S.Glasg., appointed House Surgeon to the Hulme Dispensary, Manchester. K. F. Sylvester, L.R.C.P.Edin., M.R.C.S. Eng., appointed Medical Officer of the Melksham Union Workhouse. J. S. Taggart, M.B., Ch.B.Vict., appointed House Physician to the Manchester Royal Infirmary. J. E. Tuxford, L.R.C.P., L.R.C.S.Edin., appointed Medical Officer of Health to the Boston Town Council. C. C. Vigurs, M.B., B.C.Camb., appointed Medical Officer for the No. 5 District of the St. Columb Major Union. R. J. Warrington, M.R.C.S.Eng., L.R.C.P.Lond., appointed House Physician to the Manchester Royal Infirmary. H. W. Webber, M.D., M.S.Lond., M.R.C.S.Eng., appointed Second Medical Officer to the Provident Department of the Plymouth Public Dispensary. Dr. Wilkins, appointed Medical Officer for the Third Liskeard District of the Liskeard Union. J. Wilkinson, M.B., C.M.Edin., appointed Medical Officer of Health to the Boston Rural District Council. A. P. Woollright, L.S.A.Lond., appointed Junior House Surgeon to the West Ham Hospital. A. G. Young, M.B., appointed Medical Officer for the Rillington District of the Norton (Out-Relief) Union.

### VACANCIES.

The following vacancies are announced this week, the amount of salary in each case being given after the name of the institution:—

### Resident Medical Officers.

Belgrave Hospital for Children, 77 and 79, Gloucester Street, S.W.—House Surgeon. Board, fuel, and lights provided. Applications, endorsed "House Surgeon," to the Honorary Secretary at the Hospital by September 28th.

Bethlem Hospital.—Two Resident Clinical Assistants; doubly qualified; appointment for six months. Apartments, rations, washing, and attendance provided. Applications, endorsed "Clinical Assistantship," to the Treasurer at Bethlem Hospital before October 1st.

Birmingham and Midland Eye Hospital.—Assistant House Surgeon. Salary £50 per annum, with apartments and board. Applications to the Chairman of the Medical Board by September 30th.

Durham County Asylum.—Junior Medical Officer. Salary £100 per annum, with board and lodging. Applications, with not more than three recent testimonials, to the Superintendent, Durham County Asylum, Winterton, Ferryhill, by September 28th.

Essex and Colchester Hospital.—House Surgeon, doubly qualified. Salary £80 per annum, with board and lodging in the hospital. Applications to the Committee by October 18th.

Glasgow Maternity Hospital.—Obstetric Physician and Assistant Obstetric Physician. Applications to Arthur Forbes, Secretary, 146, Buchanan Street, Glasgow, by November 8th.

London Hospital, Whitechapel, E.—Medical Electrician; must be qualified and registered under the Medical Act. Applications to G. Q. Roberts, House Governor, by October 19th.

London Fever Hospital, Liverpool Road, Islington, N.—Assistant Resident Medical Officer. Salary £120 per annum. Applications to the Secretary by October 1st.

Parish of Durness, Sutherlandshire.—Medical Officer. Guaranteed salary £150 per annum, with practice, free house, and garden. Applications to Robert Sutherland, Inspector of Poor, Durness, by October 19th.

Rotherham Hospital and Dispensary.—Assistant House Surgeon; doubly qualified, and registered. No salary, board, lodging, and washing. Applications and testimonials to the House Surgeon by October 1st.

Royal Alexandra Hospital for Sick Children, Dyke Road, Brighton.—House Surgeon; doubly qualified. Salary £80 per annum, with board, lodging, and washing; no stimulants. Applications to the Chairman of the Medical Committee by October 6th.

Sussex County Hospital, Brighton.—Assistant House Surgeon; doubly qualified, unmarried, and, when elected, under 30 years of age. Salary not exceeding £30 per annum, with board, washing, and residence in the hospital. Applications to the Secretary by October 2nd.

### Non-Resident Medical Officers.

Brighton Throat and Ear Hospital, 23, Queen's Road, Brighton.—Non resident House Surgeon. Salary at the rate of £52 per annum Applications to the Secretary by September 30th.

Carnarvon Joint Sanitary District.—Medical Officer of Health, must be between 25 and 40 years of age, doubly qualified. Must devote his whole time to the duties, and have a knowledge of the Welsh language. Appointment for five years. Salary £694 per annum, inclusive of all expenses, except those incurred for such books, stationery, and apparatus required in the performance of the duties. Applications, endorsed "Application for Office of M. O. Health," to J. H. Thomas, Clerk to the Joint Committee, 14, Market Street, Carnarvon, by October 16th.

County of Brecon.—County Analyst. Retaining fee £10 10s., and a fee of 10s. 6d. for the analysis of every sample. Applications to H. Edgar Thomas, Clerk to the County Council, County Hall, Brecon, by October 2nd.

General Hospital, Birmingham.—Two Assistant House Surgeons, must possess a surgical qualification. Appointment for six months. No salary, but residence, board, and washing provided. Applications to Howard J. Collins, House Governor, by September 28th.

Norfolk and Norwich Hospital.—Dispenser; must be registered Pharmacist. Salary £100. Applications to the Secretary by October 1st.

Samaritan Hospital for Women, Nottingham.—Junior Surgeon. Applications to the Honorary Secretary, J. A. Simpson, Solicitor, South Parade, Nottingham, by October 1st.

